# “Adult rhabdoid tumors—a riddle inside an enigma?”

**DOI:** 10.1038/s41379-022-01144-1

**Published:** 2022-09-21

**Authors:** Pascal D. Johann

**Affiliations:** Swabian Children’s Cancer Center, 86156 Augsburg, Germany

In this issue of Modern Pathology, Duan et al. (reference) present their work on primary adult sellar SMARCB1-deficient tumors. This entity represents one further group in the ever expanding universe of SMARCB1 deficient tumors:

While the most prominent entity that displays a homozygous loss of the SMARCB1 are undoubtedly rhabdoid tumors (RT), the last years have seen molecular characterizations of further (extra and intracranial) SMARCB1 or SMARCA4 deficient entities^1^ including SMARCB1 deficient chordomas^2^, cribriform neuroepithelial tumors^3^ and renal medullary carcinomas^4^.

But even when only considering RT, it is increasingly clear that differences between eMRT (extracranial malignant RT) and ATRT (Atypical teratoid RT) that may exist at the morphological and immunohistochemical level blur at the molecular level: Both the loss of SMARCB1^5^ but also a shared methylation-phenotype emerge as a unifying feature of ATRT and eMRT^6^.

**Fig. 1:**
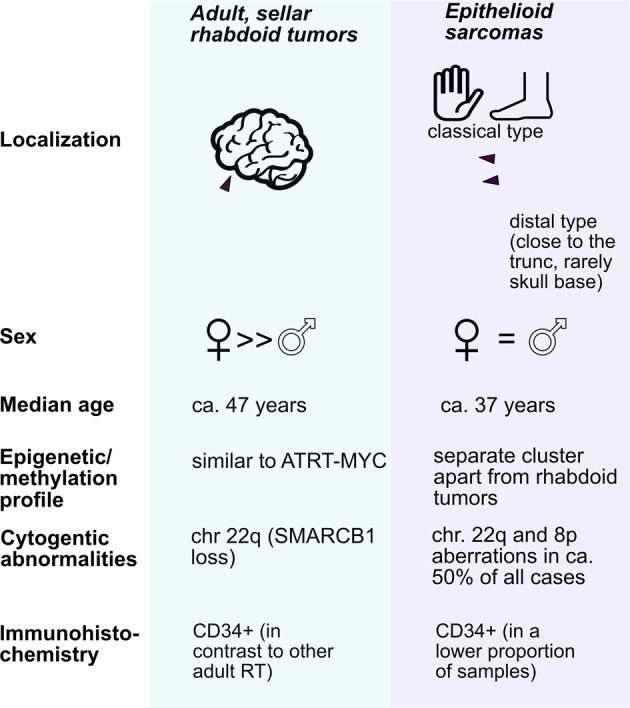
Synopsis on epithelioid sarcomas and rhabdoid tumors. An overview on important clinical and molecular characteristics in epithelioid sarcomas versus adult, sellar rhabdoid tumors.

An important differential diagnosis to RT include epithelioid sarcomas. These rare sarcomas—most of which also display a homozygous loss of SMARCB1/INI1—predominantly arise in the acral parts of the body, but can also occur in intraabdominally or very rarely even at the skull basis^7^. In contrast to RT, other cytogenetic abnormalities (such as the loss of 8p in about 50% of all cases, Fig. 1^8^), have been described.

The distinction between RT and epithelioid sarcomas is clinically relevant as the latter—unlike RT—are usually not amenable to cytostatic therapy and an early resection should be attempted.

Duan et al. investigate eight adult, sellar SMARCB1 deficient tumors and elucidate their molecular makeup, aiming at differences and commonalities to RT and epithelioid sarcomas as well as SMARCB1 deficient chordomas.

In a methylation analysis (based on EPIC arrays), these tumors cluster with the ATRT-MYC subgroup. While this similarity been described before^9^, the authors now confirm previous analysis in a relatively large cohort given the rarity of this tumor type.

They also find CD34—a marker otherwise mostly absent in adult RT—to be present in most of the tumor samples. Unfortunately, this marker is also commonly present in epithelioid sarcomas and thus unsuitable for clear, immunohistochemical distinction of both entities. With regards to a further immunohistochemical characterization, it is notable that the authors cannot confirm SALL4 and ERG to be good discriminatory markers that distinguish between Epithelioid Sarcoma and RT as it has been otherwise proposed^10^.

Overall, the publication by Duan et al. represents an important piece to define the landscape of SMARCB1 deficient tumors.

An unresolved issue that remains to be elucidated is the female predmoninance among patients with sellar adult, RT.

Along a similar line, the localization of these tumors is enigmatic: The predominance of sellar tumors among adult ATRT^11^ is intriguing given that this localization is not prominent in pediatric rhabdoid tumor series. One may speculate that these tumors may evolve from other cells of origin than pediatric RT. Some evidence (such as the lack of CD34+ in other adult RT) indeed points to sellar, adult RT as a separate nosological entity. However, a more comprehensive review that would include whole genome and transcriptomic data still remains elusive. Also, the currently published number of sellar adult rhabdoid tumor cases seems to small to reliably infer that there are not genetic lesions beyond SMARCB1.

Other clinically interesting features such as a potential endocrine dysfunction owing to the localization close to the pituitary gland remain to be investigated systematically. Also, a potential adaption of treatment strategies to the adult patient cohort remains to be implemented.

Overall, while diagnostic features of sellar, RT emerge and their methylomic similarity to ATRT-MYC becomes more and more apparent, the clinical characterization of this “riddle inside an enigma” would be an important step forward to ultimately improve the bleak prognosis for adult rhabdoid tumor patients.
